# Draft Genome Sequence of *Salmonella enterica* subsp. *enterica* Serotype Oranienburg Strain S-76, Isolated from an Aquatic Environment

**DOI:** 10.1128/genomeA.01017-13

**Published:** 2013-12-12

**Authors:** Andrés Medrano-Félix, Mitzi Estrada-Acosta, Maribel Jiménez, Bruno Gómez-Gil, Josefina León-Félix, Luis Amarillas, Cristóbal Chaidez

**Affiliations:** Laboratorio de Microbiología Ambiental y de Alimentos, Centro de Investigación en Alimentación y Desarrollo, A.C., Unidad Culiacán, Culiacán, Sinaloa, Mexicoa; Facultad de Ciencias Químico-Biológicas de la Universidad Autónoma de Sinaloa, Ciudad Universitaria, Culiacán, Sinaloa, Mexicob; Centro de Investigación en Alimentación y Desarrollo, A.C., Unidad Mazatlán, Mazatlán, Sinaloa, Mexicoc; Instituto de Investigación Lightbourn, Chihuahua, Mexicod

## Abstract

*Salmonella* is a widespread microorganism and a common causative agent of food-borne illnesses. *Salmonella enterica* subsp. *enterica* serotype Oranienburg is highly prevalent in surface water from tropical ecosystems and is not commonly related to illnesses. Here, we report the first genome sequence of *Salmonella* Oranienburg strain S-76, isolated from an aquatic environment.

## GENOME ANNOUNCEMENT

The global presence of *Salmonella* is made possible by its wide host range capability and the numerous environmental settings in which this bacterium is able to survive and multiply (1). Among its possible settings, rivers (2, 3), lakes and seawater (4), and soil and sediments (5) are considered the important ecological niches (6) where *Salmonella* is able to survive over long time periods (7, 8). Commonly, the diversity of serotypes associated with human and nonhuman illnesses differs from those serotypes that are isolated from or distributed in the environment; the latter can exert selective pressure leading to the establishment of specific serotypes based on genetic response, as well as possible changes or modifications to the genetic content. According to the NCBI (http://www.ncbi.nlm.nih.gov/nuccore/?term=salmonella+genome), the most commonly reported *Salmonella* genome sequences belong to reference strains or clinical isolates of *Salmonella enterica* subsp. *enterica* serotypes Enteritidis, Typhi, and Typhimurium, and environmental isolates are the least studied at the genomic level.

Several studies conducted by our team have described the prevalence and diversity of *Salmonella enterica* subsp. *enterica* serotype Oranienburg in the environmental settings of Culiacán, Mexico. However, to our knowledge, there are no studies targeting the environmental bacterial genome sequence to determine, at the genetic level, its adaptation and prevalence mechanisms. We report a draft genome sequence of a *Salmonella* Oranienburg collection strain (S-76) from the food and environmental microbiology laboratory at the Centro de Investigación en Alimentación y Desarrollo, Culiacán Station.

To our knowledge, this is the first report of genomic sequencing for a strain of *Salmonella* Oranienburg isolated from an aquatic environment. The genome was sequenced by the CMO-based sequencing technology, using the Ion Torrent Personal Genome Machine (PGM) and the Ion 316x chip. The next-generation sequencing process resulted in ~35.8-fold coverage (188 Mbp sequenced), with a mean read length of 92, and around 2,052,223 reads were assembled into 182 contigs (*n* = 61.5 kbp) using the MIRA software 3.4.0.

The assembled genome sequence of S-76 was processed for rapid annotation using the Rapid Annotations using Subsystems Technology (RAST) server (9); the results show the chromosome of *Salmonella* Oranienburg S-76 to be composed of 4,609,551 bp, including 5,020 coding sequences (CDSs), 103 tRNAs, and an average G+C content of 52.2%. A brief analysis of the sequenced genome reveals at least 816 genes related to carbohydrate metabolism, including 162 genes involved in central carbohydrate metabolism, such as pyruvate metabolism, the tricarboxylic acid cycle (TCA), the pentose phosphate pathway, the Entner-Doudoroff pathway, and glycolysis. Additionally, S-76 contains 188 genes involved in stress response, such as osmotic stress, desiccation, oxidative stress, cold and heat shock, and periplasmic stress response.

Another gene set of great importance is the content involving DNA metabolism; this genome contains 186 genes involved in functions such as DNA repair, DNA replication, recombination, uptake, and competence, with the DNA repair genes being the predominant gene set. Together, these genes may contribute to the adaptation and prevalence of the organism in aquatic environments.

### Nucleotide sequence accession numbers.

This whole-genome shotgun project has been deposited at DDBJ/EMBL/GenBank under the accession no. AROZ00000000. The version described in this paper is version AROZ01000000.
